# Impact of active and latent concerns about COVID-19 on attention

**DOI:** 10.1186/s41235-022-00401-w

**Published:** 2022-06-03

**Authors:** Caitlin A. Sisk, Yi Ni Toh, Jihyang Jun, Roger W. Remington, Vanessa G. Lee

**Affiliations:** 1 Department of Psychology, University of Minnesota, 75 East River Road, S504 Elliott Hall, Minneapolis, MN 55455 USA; 2 Center for Cognitive Sciences, University of Minnesota, 75 East River Road, S504 Elliott Hall, Minneapolis, MN 55455 USA

**Keywords:** COVID-19, Selective attention, Sustained attention, Emotion, Mental health

## Abstract

The interactions between emotion and attention are complex due to the multifaceted nature of attention. Adding to this complexity, the COVID-19 pandemic has altered the emotional landscape, broadly heightening health and financial concerns. Can the heightened concerns about COVID-19 impair one or more of the components of attention? To explore the connection between heightened concerns about COVID-19 and attention, in a preregistered study, we collected survey responses from 234 participants assessing levels of concerns surrounding COVID-19, followed by four psychophysics tasks hypothesized to tap into different aspects of attention: visual search, working memory, sustained attention, and cognitive control. We also measured task-unrelated thoughts. Results showed that task-unrelated thoughts, but not survey reports of concern levels, negatively correlated with sustained attention and cognitive control, while visual search and working memory remained robust to task-unrelated thoughts and survey-indicated concern levels. As a whole, these findings suggest that being concerned about COVID-19 does not interfere with cognitive function unless the concerns are active in the form of task-unrelated thoughts.

**Supplementary Information:**

The online version contains supplementary material available at 10.1186/s41235-022-00401-w.

## Significance statement

This study is the first to directly measure relationships between concerns about COVID-19 and multiple components of attention. The results show that the effects of heightened concerns on tasks requiring attention differ depending on the task demands, as well as the extent to which concerns intrude into active thought. This finding offers important insights into the kinds of daily tasks, such as driving, schooling, and workplace duties that are likely to be susceptible to interference from heightened concerns during crises like the current pandemic. The findings suggest that heightened concerns about COVID-19 do not greatly interfere with performance on tasks requiring selective or sustained attention.

## Introduction

The emergence of SARS-CoV-2 suddenly and profoundly altered the daily lives, workplace operations, and internal experiences of many people. The novel coronavirus disease 2019 (COVID-19) caused more than 500 million infections and 6.2 million deaths worldwide. Vaccination effort has ramped up, but the virus and its many variants continue to pose a threat to a large swath of the population. As the race between widespread vaccination and new variants continues, researchers are just beginning to understand the effects of the pandemic’s ever-changing landscape on human behavior. With increasing concerns and shifting priorities, many feel preoccupied with thoughts about the physical and financial threats the virus and the resulting economic upheaval may pose. A poll conducted in July 2020 found that 66% of Americans were worried about themselves or a family member being infected (Washington Post–ABC News, July 17, 2020). The elevated concerns may draw attention away from important stimuli in daily tasks, such as driving, schooling, and workplace responsibilities. Might preoccupation with the physical and financial threats of COVID-19 interfere with performance on tasks requiring attention?

To assess the potential impact of COVID-related concerns on cognitive performance, in this study we measured the correlation between levels of concern about COVID-19 and performance on several different cognitive tasks. Our goals are to (i) determine whether there is a connection between these heightened concerns and performance on tasks relying on attention, and (ii) whether some components of attention are more susceptible to interference from current concerns than others. In turn, the findings will shed light more broadly on the relationship between emotion and cognition, two research fields that have seen increasing connections in recent years (Pessoa, 2008).

Some evidence suggests that internal states, such as ongoing worries and fear, interfere with attention. Studies on mind wandering showed that people frequently engage in task-unrelated thinking in everyday activities (Killingsworth & Gilbert, 2010). These task-unrelated thoughts (TUTs) are often grounded in participants’ current concerns (McVay & Kane, 2010; Poerio et al., 2013; Smallwood & Schooler, 2015). These findings suggest that in the face of heightened concerns, the intrusion of task-irrelevant thoughts can draw attention away from ongoing tasks. In other words, severe concerns about COVID-19 may lead people to become more distractable. As yet, it is unclear whether COVID-related concerns manifest as intrusive task-unrelated thoughts or associated deficits in attention, nor whether, as with mind wandering, the relationship between attention and concerns would be affected by factors such as task difficulty or an individual’s working memory capacity (McVay & Kane, 2012; Seli et al., 2018; Smallwood & Schooler, 2015). The present study addresses whether people with greater COVID-related concerns are more prone to mind wandering than those with low concern levels, and if so, whether these concerns induce general task impairment, or whether the impairment is specific to tasks that implicate a particular attentional function.

Other studies have found that emotions, such as fear, modulate attention. Compared with neutral stimuli, fearful stimuli tend to capture attention more and are less susceptible to the attentional blink (Anderson & Phelps, 2001; Compton, 2003; Ohman et al., 2001). Fear may also narrow the focus of attention to fear-inducing stimuli, as in the “weapon-focus” effect (Steblay, 1992). However, COVID-related concerns may give rise to general anxiety, rather than specific fears. The relationship between anxiety and attention is complex (Robinson, Krimsky, et al., 2013; Robinson, Vytal, et al., 2013). For example, although patients with an anxiety disorder may have deficits in brain regions regulating cognitive control, they do not always perform more poorly on cognitive control tasks (Eysenck et al., 2007; Lagarde et al., 2010). Experimentally induced anxiety, such as the threat of receiving an electric shock in an experiment, does not consistently impair cognitive control (Choi et al., 2012; Robinson Krimsky, & Grillon, 2013; Robinson, Vytal, et al., 2013). Thus, while studies on acute emotions suggest that internal states may affect attention, studies on prolonged emotional states like anxiety suggest that individuals may suppress the cognitive effects of sustained environmental stressors during performance of cognitive tasks.

However, studies on the effects of poverty and resource scarcity argue that severe concerns do influence cognitive performance. Mani et al. (2013) showed that poverty is associated with impairments in cognitive control. In one experiment, they asked shoppers at a mall in the USA to think about a car repair. Some were told the repairs would cost $1,500, whereas others were told it would cost $150. They then completed the Raven’s Progressive Matrices and a spatial compatibility task. Performance was comparable between high- and low- income participants when contemplating the more affordable car repair, but performance was impaired when the low-income (but not high-income) participants contemplated the expensive car repair. In another experiment, seasonal sugarcane farmers from India were tested in a numerical Stroop task either before harvest, when the farmers had limited financial resources, or after harvest, when the farmers had more financial resources. Performance was worse before than after harvest. These findings suggest that severe financial concerns compete for attention with ongoing tasks.

The Mani et al. (2013) study has received some criticism regarding its methods, controls, and conclusions (Dang et al., 2015). It nonetheless sparked an interest in the effects of resource scarcity on cognitive performance. Other studies have found behavioral changes in people who are scarce in other resources, such as time (Cannon et al., 2019; Roux et al., 2015). These findings, in the context of the broader literature linking attention and emotion, raise the possibility that as access to financial or health resources declines during the COVID-19 pandemic, cognitive control may also suffer, whether due to depleted resources or increased anxiety surrounding those resources.

Two shortcomings of these prior studies may account for the mixed findings regarding the impairment of or robustness of cognitive performance in the face of anxiety or concerns. First, attention is a multifaceted construct that relies on multiple brain systems (Pashler, 1997). For example, whereas cognitive control primarily engages the prefrontal cortex (Badre, 2008; Braver, 2012), sustained attention relies on a broad neural network including the frontal, parietal, temporal, and cerebellar cortices (Esterman et al., 2013; Rosenberg et al., 2016). Yet previous studies of anxiety and attention have largely used a single index of attention. It is unclear, then, how widespread the effects are or whether there is a systematic relationship between the effects of anxiety and components of attention at various levels of cognitive processing. Secondly, measuring only “latent” concerns, without taking into account “active” concerns in the form of task-unrelated thought, results in an incomplete picture of the economics of scarcity and the effects of concerns on attention. The contents of TUTs frequently reflect an individual’s current goals (Klinger, 1971, 2013), which vary over time and across tasks (Rummel et al., 2021; Zanesco, 2020). TUTs are also sensitive to future-related concerns (Stawarczyk et al., 2013). However, people may be able to exert cognitive control over long-term concerns, rendering them “latent” rather than active. Do scarcity and the anxiety it causes consistently influence attention, or do their effects depend on the concerns being activated and emerging into conscious thought?

A comprehensive understanding of the effects of pandemic-related concerns on attention therefore requires the inclusion of multiple cognitive tasks that tap into distinct components of attention, as well as measures of both latent and active concerns. Regarding the selection of cognitive tasks, several studies have sought to identify distinctive attentional constructs that can be measured by psychophysics (Fan et al., 2002; Skogsberg et al., 2015; Treviño et al., 2021), converging on a few findings. Fan et al. (2002) measured individual differences using the Attention Network Test (ANT), a timed reaction time (RT) task that combined spatial cues with flanker interference. It provided a measure of an executive control component in the difference between congruent and incongruent flanker trials, as well as measures of orienting and alerting components in the difference between spatial cue conditions. Using hierarchical-cluster analysis across 11 different attention tasks, Skogsberg et al. (2015) identified spatiotemporal attention, global attention, transient attention, and sustained attention as the four components of visual attention. Each of the 11 tasks held a place within a 2-dimensional space that differed along the global to spatiotemporal dimension on one axis and the sustained to transient dimension on the other. More recently, Treviño et al. (2021) used six experimental tasks, including the scene CPT as a measure of sustained attention and five selective attention tasks (multiple object tracking, spatial configuration visual search, visual working memory, approximate number sense, and flanker interference). They identified five components of attention based on exploratory factor analysis: capacity, search, digit span, arithmetic, and sustained.

Although these studies differed in terms of the tasks used and components identified, there were some consistencies. First, sustained attention featured in each set of components, represented as alerting in Fan et al. (2002), and sustained attention in Skogsberg et al. (2015) and Treviño et al. (2021). Second, spatial orienting consistently appeared as a component, termed orienting in Fan et al. (2002), spatiotemporal attention in Skogsberg et al. (2015), and search in Treviño et al. (2021). Third, two of the three included a component related to executive function or cognitive control. Fan et al. (2002) identified an executive control component, and although Treviño et al. (2021) did not identify cognitive control as a specific component, their arithmetic component is related to cognitive control. Most importantly, all three studies divided the general cognitive function of attention into multiple separable components.

Of the different components identified by these studies, some are more externally driven (e.g., search in Treviño et al., 2021 or spatiotemporal attention in Skogsberg et al., 2015), while others are more internally oriented (e.g., executive control in Fan et al., 2002 or arithmetic in Treviño et al, 2021). The different components of attention may vary along a scale from external, or perceptual, to internal, or central, attention (Chun et al., 2011). Tasks whose performance relies on external, visual stimuli are more perceptual in nature, whereas tasks whose performance relies on internal regulation of rules and responses are more closely tied to central attentional functions.

Regarding the evaluation of both latent and active concerns, a recent sustained attention study (Jun et al., 2021) provided evidence of the importance of this distinction. Jun et al. (2021) was among the first to directly evaluate the effects of concerns about COVID-19 on attention. The study was conducted in the first year of the pandemic before vaccines became widely available. Young adults from Europe and the USA first rated their levels of health-related and financial concerns surrounding the COVID-19 pandemic. They then completed a demanding continuous performance task (CPT), withholding response to infrequent mountain images presented among a stream of city images. Participants also self-reported the proportion of the time that their mind had wandered from the task. Despite expressing a wide range of COVID-related concerns in the pre-task questionnaire, participants with severe concerns did just as well on the scene CPT as participants with low concerns. However, those experiencing a higher rate of mind wandering during the scene CPT did more poorly. Jun et al. (2021) suggest that young adults are largely successful in preventing their pre-task COVID concerns from intruding into the scene CPT. TUTs that did occur during the task, however, became a source of distraction that interfered with performance. This finding underscores the need to consider two types of concerns: concerns that people can regulate and minimize during a task (“latent concerns”) and concerns that intrude into a task (“active concerns”). Whereas latent concerns can be measured using a pre-task questionnaire, active concerns manifest as active TUTs during the task. Because Jun et al. (2021) tested a single attentional construct—sustained attention—it is unclear whether effects of current concerns on attention are broad, or whether they are limited to certain attentional functions.

**Fig. 1:**
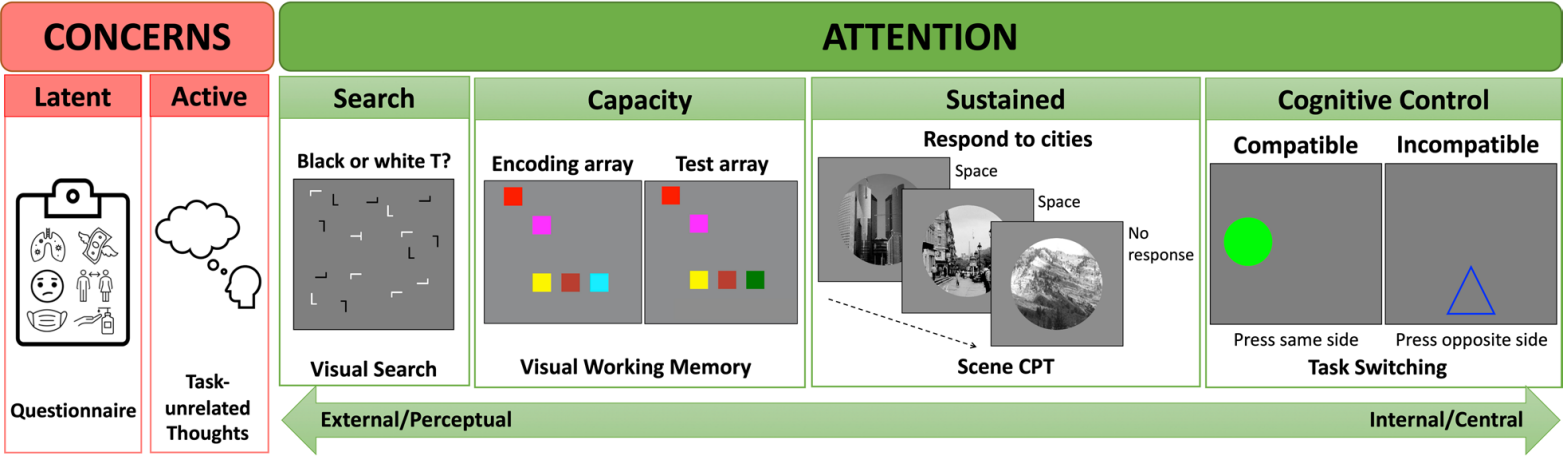
A schematic of the tasks. Left. A pre-task questionnaire asked participants to rate their level of concerns about COVID-19. Participants also estimated the rate of task-unrelated thoughts (TUTs) at the completion of each cognitive task. Right. The four attention tasks included visual search, visual working memory, scene CPT (sustained attention), and task switching. Participants were tested in one of four orders of the tasks based on a balanced Latin Square design

In this preregistered study, we go beyond the single-task assessment of Jun et al. (2021) in pursuit of two main goals: (1) to test the effects of reported COVID-related concerns on multiple components of attention, and (2) to investigate whether task-unrelated thoughts primarily influence externally or internally driven components of attention. We collected data from young adults during the COVID-19 pandemic from October 2020 to February 2021, before vaccines for COVID-19 became widely accessible (https://osf.io/5y9bt/?view_only=615432100a084e2faf535942d1015073). We tested 234 participants in four attention tasks that probe different components of attention: visual search, visual working memory, sustained attention (using a scene CPT), and task switching. Immediately before the attention tasks, participants completed a questionnaire assessing their level of health concerns and financial concerns related to COVID-19 (Jun et al., 2021). They also completed a questionnaire using the State-Trait Anxiety Inventory (STAI-6; Spielberger, 1983; Tluczek et al., 2009) to measure anxiety. These questionnaires are taken as a measure of latent concerns. After each attention task, participants reported the proportion of time spent on TUT during the task. The TUT reports are taken as a proxy for active concerns. We test the prediction that elevated active concerns, reflected in the TUT measure, are associated with impaired attention, especially for central attention components, rather than perceptual attention components. Here, we provide an overview of the four attention tasks (Fig. 1).

### Visual search

The visual search task varied in two factors: nature of search (feature or conjunction search) and the number of items (i.e., set size of 8 or 16). This task loaded onto the search component of attention in Treviño et al. (2021) and is closely related to the orienting and spatiotemporal components identified in Fan et al. (2002) and Skogsberg et al. (2015), respectively. Participants searched for a letter T among distractor letters and reported whether the T was black or white. In the feature search condition, the distractors were Os, yielding a large, featural difference between the target and the distractors. In the conjunction search condition, also known as the spatial configuration search condition, the distractors were Ls, yielding a small, spatial configurational difference between the target and distractors (Wolfe, 1998). The slope of the linear function relating RT to set size indexes search efficiency. Feature search produces shallow search slopes, owing to the availability of preattentive feature information guiding search quickly to the target (Treisman, 1996; Wolfe, 2021). But conjunction or spatial configuration search cannot rely on the same preattentive information and therefore yields steep search slopes that provide an index of search efficiency.

### Visual working memory

Many people categorize visual working memory as an example of attention directed internally (Chun et al., 2011), requiring some cognitive control. To assess visual working memory, we adapted a standard color visual working memory task (Xie et al., 2020) using the change detection procedure. This task loaded onto the capacity component in Treviño et al. (2021). Participants encoded to memory a display of 5 color patches that were presented for 0.5 s. After a blank delay interval of 1.5 s, a test display of 5 colors was presented in the same locations, including four colors that were the same as before and one that had changed. Participants were asked to click on the color that had changed. Because the task requires a comparison between the test array and an internally maintained memory representation, our preregistered predictions were based on the assumption that the visual working memory task relies primarily on central attention. However, the change detection task does not require manipulation of information in visual working memory (Pailian & Alvarez, 2020); as such, it may be more robust to current concerns than other versions that require information manipulation.

### Scene CPT

As a continuous performance task, the scene CPT presented participants with a continuous stream of city and mountain images (800 ms/scene) for two minutes (Esterman et al., 2013; Jun et al., 2021). It taps into the sustained attention component identified in previous studies. In our study, participants pressed the spacebar to cities that occurred on 90% of the trials and withheld response to mountains that occurred on 10% of the trials. The mountains and cities were not visually salient, nor did the two differ from one another in salient ways, reducing bottom-up signals that could be used for detection. Although CPT tasks are commonly used to index sustained attention, performance relies on multiple mechanisms, including perceptual processing, rhythmic responses to the onset of the stimuli (Hawkins et al., 2019), and inhibitory control of responses (Jun & Lee, 2021).

### Task-switching

As a measure of cognitive control, we adapted a version of the task-switching paradigm used by Mani et al. (2013). This task involves both congruent and incongruent trials, similar to the flanker aspect of Fan et al. (2002) that was used to assess executive function. The task also requires memory of and application of task rules, which likely taps into similar mechanisms to those categorized in Treviño et al. (2021)’s arithmetic component. In this task, a geometric shape appeared in one of four locations around the center (top, bottom, left, or right). The participants’ task was to report its location using arrow keys on their keyboard. The response rule was either compatible–pressing the arrow corresponding to the location of the shape, or incompatible–pressing the arrow opposite to the location of the shape, which incorporated a Stroop-like congruency effect. In *task-stay* blocks, the same rule was used for a block of trials. In *task-switch* blocks, the two rules were randomly intermixed within a block of trials: outline shapes required the incompatible mapping, but solid shapes required the compatible mapping. Following Mani et al. (2013), we used accuracy in the task-switch blocks as an index of performance. Because the complexity of this task-switching paradigm largely originates from a change in response rules, this task relies primarily on central attention.

Together, visual search, visual working memory, scene CPT, and task switching capture a variety of attentional mechanisms (Treviño et al., 2021), allowing us to assess the potentially different effects of current concerns on different aspects of attention.

## Method

### Preregistration

This study was preregistered on the Open Science Framework (https://osf.io/5y9bt/?view_only=615432100a084e2faf535942d1015073). The preregistration included details about the study purpose, hypotheses, sample size determination, design, and analysis plan.

### Participants

#### Sample size determination

G*Power analysis (Faul et al., 2007) was used to estimate a minimum sample size that corresponds to a power greater than 0.95 for detecting a significant correlation between current concerns and attention task performance using a two-tailed test. In this power analysis, we applied a moderate effect size (*ρ* = 0.3) and a Bonferroni-corrected critical alpha level of 0.002 (which allowed for up to 25 planned tests). The selection of a moderate effect size in our power analysis was based on the effect sizes reported in two recent studies that have explored the effects of COVID-related concerns on task performance in the CPT used here (Jun & Lee, 2021) and a working memory task (Xie et al., 2020), both of which showed effect sizes in this range. The power analysis showed that the minimum sample size was 232. Counterbalancing of task orders required that participants be tested in multiples of four. We therefore planned to reach a final sample size of 232 participants after any exclusions.

Two participants who were initially excluded were later found to meet the inclusion criteria and were therefore added back to the analyzed sample, resulting in a final sample size of 234 participants, including 134 males, 93 females, and 7 participants who did not identify as male or female. The mean age of those included in the final dataset was 29.6 years (range 18–45; SD = 7.4). Participants were recruited from Prolific.co, an online website for behavioral research. They met the inclusion criteria: 18–45 years of age; fluent English; current residence in the USA; normal vision; and no history of neurological or psychiatric conditions. They provided informed consent through Qualtrics and received monetary compensation. The study was approved by the University of Minnesota’s Institutional Review Board. Additional demographic information can be found in Additional file 1: Table S2.

#### Data exclusion

Data from 23 additional participants were excluded according to preregistered data exclusion criteria. Four participants were excluded for accuracy below 80% in the visual search task. Six participants were excluded for responding to fewer than 30% of the “go” trials in the scene CPT. One participant was excluded for below chance (25%) accuracy in task switching. And one participant was excluded for meeting the exclusion criteria in visual working memory (< 20% accuracy), scene CPT, and task switching. That participant also met the survey-based exclusion criterion of selecting the incorrect response when directly asked to choose “2” as their response on the Likert scale. Finally, eleven participants were excluded because they had previously completed a nearly identical CPT task and therefore lacked the required naivete.

### Procedure

Participants first completed a COVID-19 survey through Qualtrics. Informed consent was administered via Qualtrics prior to the presentation of survey questions. Questions pertaining to inclusion and exclusion criteria were also presented prior to other survey questions, and participants who did not qualify were not allowed to continue.

#### COVID-19 survey

We developed the survey based on existing COVID-19 surveys (Conway et al., 2020; Grasso et al., 2020) and the PhenX toolkit (phenxtoolkit.org/covid19). It began with demographic questions, followed by questions about COVID-19 infection history and Likert scale questions where participants rated their levels of concerns about the physical and financial threats posed by COVID-19 and the resulting pandemic. The survey also assessed general anxiety, adherence to public health recommendations, physical risk based on occupation and preexisting health conditions, and financial risk based on financial stability. When the first 109 participants were tested, vaccines for COVID-19 were not yet available. When the final 125 participants were tested, vaccines had begun to be administered. Following an update to IRB protocol, the final 89 participants were asked to report their vaccination status in addition to the other survey questions. Of those 89 participants, 24 reported being at least partially vaccinated.

The items assessing current concerns about COVID-19 included three health-related items and two finance-related items. Participants were instructed to review statements about potential scenarios that might occur as a result of the COVID-19 pandemic and indicate their level of concern about each scenario. Responses were recorded on a 7-point scale, ranging from “I am not concerned at all about this possibility” (1) to “I am extremely concerned about this possibility” (7). There was also an attention check asking participants to respond “2.” Table 1 lists the relevant items assessing current concerns. The full survey can be found in Additional file 1: Table S1.

**Table 1:** Survey items used to assess current concerns. Ratings were obtained on a 7-point scale

Item category	Item content
Health-related concerns	I or people I love may get sick from COVID-19
	COVID-19 may delay the treatment of other illnesses that I or people I love may have
	Someone that I interact with from outside of my household may infect me with COVID-19
Finance-related concerns	I may lose job-related income due to COVID-19 (i.e., a pay cut or decrease in business)
	I may lose my job due to COVID-19
Attention check	Please choose “2”

Following the survey, participants were redirected to Pavlovia.org where they completed the four attention tasks. The order of the tasks was determined using a Balanced Latin Square Design. The Balanced Latin Square Design protects against order effects without requiring every possible order to be included in testing. With a Balanced Latin Square Design, there were four different task orders. Each task may be represented as a letter: A (visual search), B (scene CPT), C (visual working memory), and D (task switching). The four task orders that satisfied the Balanced Latin Square were ABDC, BCAD, CDBA, and DACB. The online testing platform that assigned participants to conditions did so with some irregularities, resulting in 58 participants being assigned to order ABDC, 59 to BCAD, 57 to CDBA, and 60 to DACB. Task order did not significantly influence any of the concern or attention measures when correcting for multiple comparisons. Trial order within a task block was pre-randomized. To minimize the possible effect of different randomizations on individual participants’ performance, the same random trial order within each task was used for all participants. Each task began with instructions for that task, followed by a short practice, and then the actual task.

#### Visual search

Participants in the visual search task searched for a target letter T presented among distractor letters (either letter Os or letter Ls). Each display contained either 8 or 16 items, half of which were in white, the other half in black. Each letter subtended approximately 40 × 40 pixels and was rotated 0, 90, 180, or 270 degrees. The letters occupied randomly selected locations in a 10 × 10 invisible matrix that subtended 500 × 500 pixels, with the constraint that the letters and their colors were evenly distributed across the four visual quadrants (e.g., with 16 items, there would be 4 items per quadrant—2 white and 2 black).

Each trial began with a central fixation cross for 500 ms, followed by the onset of the search display. There was only one T on each display, and it was equally likely to be black or white. The participants’ task was to find the letter T and press the “b” button if it was black or the “w” button if it was white. The search display was erased upon the button press response. Each correct response was followed by a green word “Correct!” for 200 ms. An incorrect response was followed by the feedback “Incorrect, try to be accurate!” displayed in red for 500 ms. The experiment started with 8 trials of practice, followed by 48 experimental trials. On feature search trials, the distractors were Os, making the target the only item with straight lines. On conjunction or spatial configuration search trials, the distractors were Ls that resembled the letter T. Figure 1 shows a sample conjunction search display. The 48 experimental trials were randomly and evenly divided into 2 set sizes (8 or 16 total items) and 2 search types (feature or conjunction search). To ensure that individual differences could not be attributed to accidental characteristics of a display or trial sequence, all participants saw the same search displays presented in the same pre-randomized order.

#### Visual working memory

Participants completed a color change detection task modeled after Xie et al. (2020). Each trial of this task began with a central fixation cross for 500 ms, followed by the encoding display of five colored squares for 500 ms. These five colors were distinct from each other and were presented in randomly selected locations within an invisible 4 × 4 matrix that subtended 440 × 440 pixels. Each square subtended 70 × 70 pixels. After a blank interval of 1.5 s, a test display of five colored squares appeared in the same locations as the encoding display (Fig. 1). One of the five squares changed to a new color not presented before, whereas the other four squares maintained their colors. The participants’ task was to click on the square that had changed color. The mouse click erased the testing display. Participants received accuracy feedback (“Incorrect” in red text for 500 ms or “Correct” in green text for 200 ms). Mouse clicks that fell within 40 pixels of the center of the square were considered correct. Participants completed 5 practice trials and 24 experimental trials. All participants saw the same randomly generated displays presented in the same pre-randomized order.

#### Scene CPT

In the scene CPT, participants first viewed a set of 10 city images and pressed “c” after each one. They were then shown 10 mountain images for 1 s each and asked not to respond. The scenes were grayscale and circular (radius = 128 pixels). After this familiarization phase, participants practiced the task. This task presented participants with a stream of scenes at a pace of 800 ms/scene. Each scene was presented for 560 ms, followed by a 240 ms blank. Participants were asked to press “c” in response to cities and withhold response to mountains (Fig. 1). The ratio of city to mountain trials was 9:1. To ensure that participants understood the task, feedback was provided during practice, reminding participants to press “c” if they missed a city, or to withhold a response if they responded to a mountain. Practice ended after 30 correct responses.

Following practice, participants completed the scene CPT without feedback for two minutes, including 150 trials. The sequence of 150 images was randomly composed using the set of 10 cities and 10 mountains, with the constraints that (i) cities comprised 90% of the trials, (ii) a specific image did not occur consecutively, and (iii) the longest run of cities (without a mountain) did not exceed 25.

#### Task switching

This task was modified from Mani et al. (2013). Participants saw displays containing either a solid geometric figure or an outline geometric figure (180 × 180 pixels in size) occurring in one of the four positions of the screen (3, 6, 9, or 12 o’clock; the center of the shape was 200 pixels from the center of the screen). Participants were asked to report the location of the figure by pressing one of the arrow keys (up, down, left, or right) following a response rule (Fig. 1). Solid figures were described as coming from the “normal world,” requiring participants to press the key corresponding to the location of the figure. Outline figures were described as coming from the “opposite world,” requiring participants to press the key opposite to the location of the shape (e.g., press the down arrow key if the figure was at the top of the screen). Participants completed three types of task blocks that differed in their response rules: compatible stimulus–response mapping for solid figures, incompatible stimulus–response mapping for outline figures, and mixed blocks of solid and outline figures that required task switching from trial to trial.

Participants practiced for each type of task block for 8 trials, starting with the compatible mapping block (“normal world”), followed by the incompatible mapping block (“opposite world”) and the mixed mapping block (“mixed world”). Practice trials started with a fixation cross (20 × 20 pixels) that lasted for 200 ms. Participants saw a geometric shape (180 × 180 pixels) that remained until a response was made. On each trial, the geometric figure could be a circle, a hexagon, an octagon, a star, a triangle, or a square. Each shape appeared equally often. Solid figures were filled with red, blue, pink, or green, with each color appearing an equal number of times. Outline figures were not filled, but the color of the outline was also randomly chosen from one of the four colors. For practice, the display remained until a response was made. Participants were given accuracy feedback for each practice trial (“Correct!” in green for 200 ms or “Incorrect, please try to be more accurate!” in red for 500 ms). The next trial started after 100 ms of blank display.

There were also three types of task blocks in the main part of the experiment: a block of 12 trials in which all shapes were solid (“normal world”), a block of 12 trials in which all shapes were outlined (“opposite world”), and a block of 24 trials in which half of the shapes were solid and the other half were outlined, presented in a randomly mixed order (“mixed world”). Participants saw each type of task block twice, so there were 6 blocks for a total of 96 trials, presented in the counterbalanced order of Normal→Opposite→Mixed→Mixed→Opposite→Normal. The geometric figure either remained until a response was made or for 1.2 s if participants did not make a response by then. In the latter scenario, the geometric figure would disappear, but the next trial would not begin until a response was made. Participants still received accuracy feedback after each response, and the next trial started after 100 ms of blank display.

#### Task-unrelated thought probes

After each of the four tasks, participants were asked to report the percentage of time during the just-completed task that they spent thinking about something not related to the task itself. To report TUT, participants clicked on a continuous response scale (0–100%). After participants had completed all four attention tasks, along with each task’s TUT probe, participants were asked to report the percentage of time across all four tasks that they spent thinking about COVID-19. Again, they reported this percentage by clicking on a continuous response scale labeled from 0 to 100%.

### Data analysis

We followed the preregistered analysis plan. De-identified experimental data in an aggregated format are available at https://osf.io/5y9bt/?view_only=615432100a084e2faf535942d1015073.

#### Primary variables

For the survey, there were two main indices of preexisting or latent concern levels. First, for pandemic-related concerns, we averaged the Likert scale scores across the five survey items assessing health and financial concerns surrounding COVID-19. Higher scores closer to 7 would indicate high levels of concern, and lower scores closer to 1 would indicate low levels of concern. In some analyses, we separated health and financial concerns. This is noted in the results when applicable. This separation was supported by findings from a factor analysis that observed separate factors for health and financial concerns surrounding COVID-19 (Jun et al., 2021). The second survey index for latent concerns was STAI-6 score. The average STAI-6 score measures anxiety level using a four-point Likert scale (Marteau & Bekker, 1992). The score is calculated by dividing the sum of three negatively keyed items and the reversed scores of three positively keyed items by 6 then multiplying that number by 20. This results in a range from 20 to 80 with higher scores indicating greater anxiety.

To measure active concerns, we also used two indices, both derived from the TUT reports provided after the attention tasks. First, we measured general (i.e., not specific to COVID-19) active concerns via each task’s post-task TUT probe. Second, we measured COVID-related active concerns via the COVID-19 TUT probe at the end of the entire attention task portion of the experiment.

Each of the attention tasks also had its own primary performance measure. In visual search, we measured performance by the search slope in conjunction or spatial configuration search trials. This was calculated by taking the difference in RT between conjunction search trials with 15 distractors and conjunction search trials with seven distractors and dividing that difference by eight (the difference in the number of distractors). Shallower search slopes indicate a more efficient search. In visual working memory, performance was indexed by average accuracy. In the scene CPT, performance was indexed by *A′*, a nonparametric measure that combines hits and false alarms (Grier, 1971). Higher *A′* indicates better performance. In task switching, we preregistered two performance measures. First and foremost, we followed Mani et al. (2013) by using average accuracy in the task-switch blocks (i.e., “mixed” world blocks) to index performance. Second, we calculated the normed RT difference between task-stay blocks (i.e., “normal” and “opposite” world blocks) and task-switch blocks (i.e., “mixed” world blocks) as a measure of task-switching cost.

#### Analyses

There were five primary planned analyses.

First, to evaluate the connections between the four tasks, we planned to run Pearson’s bivariate correlations across each of the performance indices. We made inferences using *p*-values with an alpha level of 0.0083, adjusted for multiple (6) comparisons.

Second, we planned to evaluate the relationship between latent and active concerns by measuring the correlation between the survey measures of latent concerns (average COVID-related Likert score and STAI-6 score) and the corresponding TUT measures of active concerns (COVID-related TUT report and average of post-task general TUT report, respectively). Our preregistration specified the use of a Pearson’s bivariate correlation test to evaluate the relationship between survey results and TUT reports. However, after evaluating the data, we determined that the self-reported COVID-related TUT values were drastically skewed (skewness of 3.91 [*SE* = 0.16]). This violates the assumptions of a Pearson’s correlation test. Following Jun et al. (2021), we substituted a Spearman’s correlation test in all analyses involving self-reported TUT measures. Because the correlation tests were directional and there were two of them, the critical alpha level after Bonferroni correction is 0.05.

Third, to assess the relationship between latent concerns and attention, we planned a Pearson’s correlation test between each of the task performance measures and both health and financial concerns related to COVID-19. For this and all remaining tests, the critical alpha level was 0.0033 adjusted for up to 15 multiple comparisons.^1^

Fourth, to assess the relationship between active concerns and attention, we planned a Pearson’s correlation test between each task performance measure and the TUT percentage reported after that task, in addition to the COVID-related TUT reported after the completion of all four tasks. As noted above, this was changed to a Spearman’s correlation test to account for the skewness of the TUT data.

Finally, to assess the relationship between active concerns, latent concerns, and attention, we planned a stepwise regression between each attention index and (i) latent health concerns, (ii) latent financial concerns, and (iii) active concerns (TUT value in the corresponding task). All statistics reported below relied on two-tailed tests unless otherwise noted.

## Results

Following the preregistered analysis plan, we first characterize performance on the four attention tasks and present the questionnaire results before relating the different tasks and measures to one another.

### Attention tasks

#### Visual search

##### Accuracy

Participants achieved high accuracy in the visual search task. Mean accuracy was 97.5% (S.E. = 0.3%) for set size eight and 99.0% (S.E. = 0.2%) for set size 16 in the feature search task. In the conjunction or spatial configuration search task, accuracy was 95.6% (S.E. = 0.4%) and 94.1% (S.E. = 0.6%) for set sizes 8 and 16, respectively. An ANOVA on search type and set size revealed a significant effect of search type, *F*(1, 233) = 75.67, *p* < 0.001, *η*_*p*_^*2*^ = 0.25, with higher accuracy in feature search trials than conjunction search trials. There was no significant effect of set size, *F* < 1, but there was a significant interaction between difficulty and set size, *F*(1, 233) = 15.73, *p* < 0.001, *η*_*p*_^*2*^ = 0.06, with set size having an opposite effect on accuracy between feature and conjunction trial types. We removed incorrect trials from the RT analyses.

##### RT and search slope

**Fig. 2:**
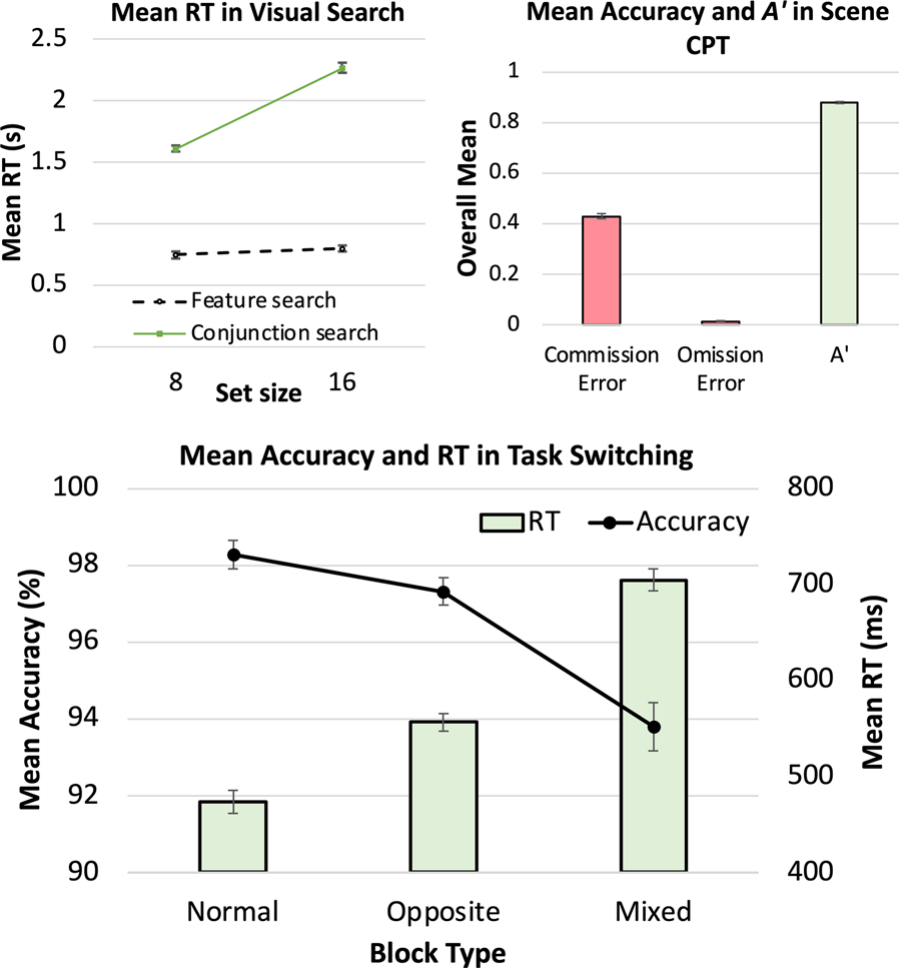
Results from the attention tasks. Upper left: Mean visual search RT. Upper right: Scene CPT accuracy and *A′*. Lower panel: Mean accuracy (%) and RT (ms) in task switching for each block type. Accuracy is plotted on the line graph corresponding to the y-axis on the left. RT is plotted on the bar graph corresponding to the y-axis on the right. Not plotted is the mean visual working memory accuracy of 58.6% (S.E. = 1.0%). Error bars show ± 1 S.E. of the mean

Figure 2 (upper left) shows the mean search RT across set sizes 8 and 16. The feature search task produced a shallow search slope of 6.5 ms/item (S.E. = 0.8 ms/item). The conjunction search task produced a steep search slope of 81.9 ms/item (S.E. = 3.9 ms/item), in line with the characterization of the task as attentionally demanding (Treisman, 1988) or inefficient (Wolfe, 1998). An ANOVA on search type and set size revealed significant effects of search type, *F*(1, 233) = 2122.29, *p* < 0.001, *η*_*p*_^*2*^ = 0.90, and set size, *F*(1, 233) = 504.14, *p* < 0.001, *η*_*p*_^*2*^ = 0.68, and a significant interaction between search type and set size, *F*(1, 233) = 346.89, *p* < 0.001, *η*_*p*_^*2*^ = 0.60, with a larger effect of set size in conjunction search than feature search. These results are in line with typical visual search findings, suggesting that the online data collection format used in the present study provided effective and reliable measurements of standard visual search behaviors.

#### Visual working memory

##### Accuracy and memory capacity

Mean accuracy in the visual working memory task was 58.6% (S.E. = 1.0%), which was significantly above the chance level of 20%, *t*(233) = 36.87, *p* < 0.001, Cohen’s *d* = 2.41. We calculated memory capacity, *K,* according to the formula used by Xie et al. (2020), where *K* = [(proportion correct × set size) − 1]. The estimated *K* of 2 was comparable to that reported previously (Xie et al., 2020), suggesting that our online data collection format effectively measured visual working memory.

#### Scene CPT

##### Accuracy and *A′* (Fig. 2, Upper Right)

Errors in the scene CPT were of two types: failing to respond to the frequent city images (omission errors) and incorrectly responding to the infrequent mountain images (commission errors). Consistent with previous studies that require participants to make a Go response frequently, errors in our study were primarily that of commission. The omission error rate was just 1.4%, whereas the commission error rate reached 43.0%, meaning that participants failed to withhold response on nearly half of the mountain (No-go) trials. Combining these two types of errors yielded a sensitivity measure, *A′*, with a mean of 0.88. (In *A′,* 0.5 is chance and 1.0 is perfect.) This performance is similar to that reported in Jun et al. (2021), reinforcing the reliability of this measure.

To confirm that our short, 2-min-long task was a valid measure of sustained attention, we evaluated the time-on-task effect. Participants’ CPT performance declined within the 2-min CPT task. *A′* in the first minute (M = 0.90, SE = 0.01) was significantly higher than *A′* in the last minute (M = 0.87, SE = 0.01), *t*(233) = 5.78, *p* < 0.001, Cohen’s *d* = 0.38.

#### Task switching (Fig. 2, Lower Panel)

##### Accuracy

The average accuracy of the two task-stay blocks was higher (M = 97.8%, SE = 0.3%) than that of the mixed blocks (M = 93.8%; SE = 0.6%), *t*(233) = 6.78, *p* < 0.001, Cohen’s *d* = 0.44, suggesting that the mixed blocks were more attentionally demanding than the task-stay blocks. For the two task-stay blocks, compatible mapping was associated with higher accuracy (M = 98.3%, SE = 0.4%) than incompatible mapping (M = 97.3%, SE = 0.4%), *t*(233) = 3.07, *p* = 0.002, Cohen’s *d* = 0.20.

##### RT

We analyzed participants’ RT after excluding incorrect trials and trials with RTs exceeding 3.5 standard deviations of each participant’s response from a given block type. Average of RT in the two task-stay blocks was faster (M = 515 ms, S.E. = 10 ms) than average RT in mixed blocks (M = 705 ms, S.E. = 11 ms), *t*(233) = 37.59, *p* < 0.001, Cohen’s *d* = 2.46, suggesting that the task-stay blocks were less attentionally demanding than the mixed blocks. In addition, compatible mapping was associated with faster RT (M = 473 ms, S.E. = 12 ms) than incompatible mapping (M = 556 ms, S.E. = 9 ms), *t*(233) = 12.04, *p* < 0.001, Cohen’s *d* = 0.79. The task-switching cost was 38.45% (S.E. = 0.9%).

In the mixed blocks, each trial is classified as either a “stay” or a “switch” trial, depending on whether it uses the same response rule as the preceding trial. RT on switch trials (M = 704 ms, S.E. = 11 ms) was slower than RT on stay trials (M = 691 ms, SE = 14 ms), *t*(233) = 2.21, *p* = 0.03, Cohen’s *d* = 0.14, demonstrating a small but significant trial-by-trial task-switching cost. The lower accuracy and longer RT in the mixed blocks compared to the task-stay blocks indicate that this task was attentionally demanding and that performance in the mixed blocks measures one’s ability to handle the cognitive control demands of task switching.

### Pre-task COVID-19 questionnaires

#### Latent concerns

##### COVID-related latent concerns

**Fig. 3:**
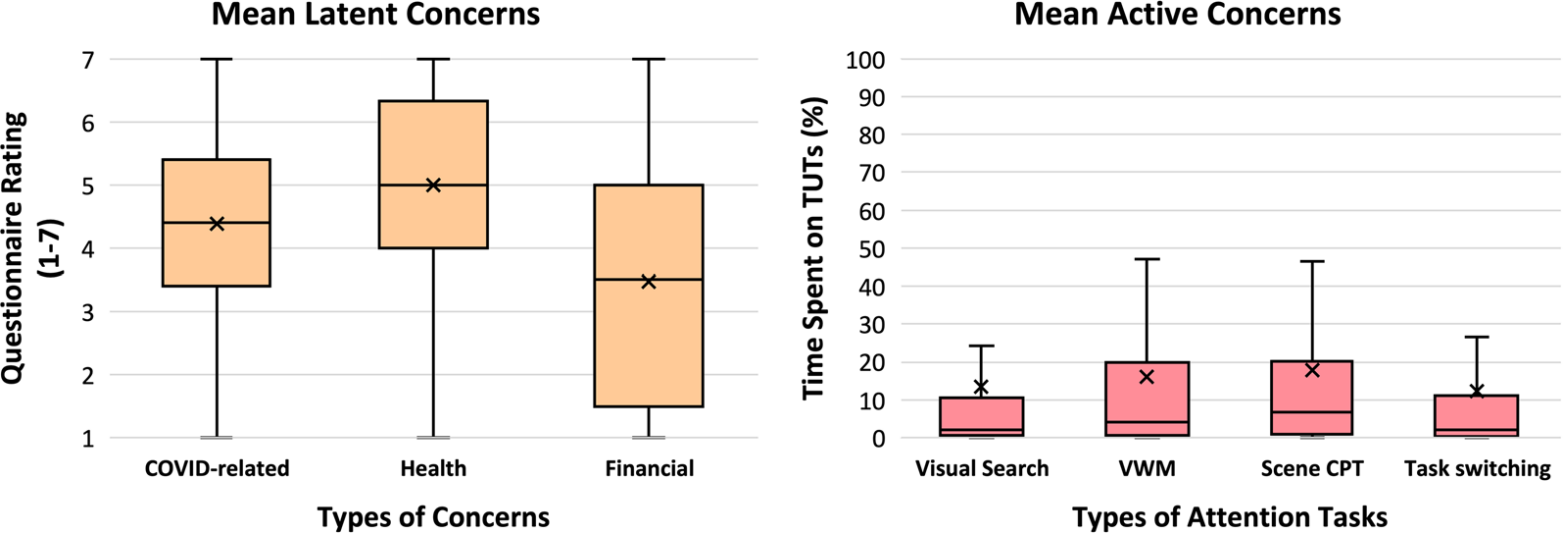
Left: Box and whisker plot of ratings on survey questions assessing latent concerns regarding COVID-19. Average across all five items is presented on the left, average of the three health-related items is presented in the middle, and average of the two finance-related items on the right. Scores closer to 7 indicate more severe concerns, scores closer to 1 indicate very low levels of concern, and scores around 4 indicate moderate levels of concern. Each box plots data from the first quartile to the third quartile. Right: Box and whisker plot of the percentage of time spent on general task-unrelated thoughts during the four attention tasks: Visual search, visual working memory, scene CPT, and task switching. Ratings closer to 100% indicate more time spent mind wandering, and ratings closer to 0% indicate full attention on the task. Each box plots data from the first quartile to the third quartile

The average rating on a 7-point scale was 4.38 (S.E. = 0.09), indicating moderate concerns (Fig. 3, left). The ratings ranged from 1 to 7 with a median score of 4.40, so participants’ COVID-related concerns ranged from mild to severe.

##### General latent concerns or anxiety

Our participants showed moderate levels of anxiety, with a mean STAI-6 score of 41.05 (S.E. = 0.96). Scores among participants ranged from 20 to 80, with a median score of 40. Thus, the level of anxiety varied across participants.

#### Active concerns

##### General task-unrelated thoughts

On average across all four tasks, participants reported spending 14.9% (S.E. = 1.5%) of the time on TUT, with a median of 5.1%, indicating that participants generally spent little time mind wandering (Fig. 3, right). In the visual search task, participants reported spending on average 13.4% (S.E. = 1.6%) of the time on TUT, median 2.1%. In the visual working memory task, the average was 16.1% (S.E. = 1.6%), median 4.1%. In the scene CPT, the average was 17.8% (S.E. = 1.7%), median 6.7%. Finally, in task switching, the average was 12.3% (S.E. = 1.5%), median 2.1%. Although the TUT rates varied across the four tasks, *F*(3, 699) = 12.40, *p* < 0.001, *η*_*p*_^*2*^ = 0.05, these values were all strongly correlated with one another, smallest two-tailed Spearman’s *rho* = 0.65, all *p*s < 0.001, meaning that participants who let their thoughts wander during one task were more likely to let their mind wander on other tasks. The median TUT and mean TUT are dissimilar, which reflects the fact that TUT was strongly positively skewed.

##### COVID-related task-unrelated thoughts

On average, participants reported spending 6.0% (S.E. = 1.1%) of the time thinking about COVID during the attention tasks, with a median of 0.4%. This value ranged from 0 to 99.5%.

### Relationships among tasks, among concern measures, and between tasks and measures

#### Correlations between the four attention tasks

Pearson’s bivariate correlations between each of the task performance measures yielded only one significant correlation between visual working memory accuracy and accuracy in the mixed blocks of the task-switching task, *r* = 0.28, *p* < 0.001, consistent with the idea that the attention tasks tapped into largely independent components (Treviño et al., 2021). In other words, people with higher visual working memory performance also had better task-switching performance. Neither visual search conjunction search slope nor scene CPT *A′* significantly correlated with performance on any other task. See Table 2 for all correlation values.

**Table 2:** Pearson’s correlation coefficients between the four attention task performance measures

Attention task	Visual search	Visual working memory	Scene CPT	Task switching
Visual search	–			
Visual working memory	− 0.06	–		
Scene CPT	0.05	0.09	–	
Task switching (mixed-block accuracy)	− 0.09	0.28*	0.10	–

Bold indicates uncorrected *p* < .01

Although we had two indices of task-switching performance—mixed-block accuracy and a task-switching cost in RT—these two indices did not correlate with each other at the adjusted alpha level, *r* = 0.11, *p* = 0.042. For this reason, further analysis relied primarily on mixed-block accuracy, the performance index used in Mani et al. (2013)

*indicates values significant at the corrected alpha level of *p* < 0.0083

##### Relationship between active and latent concerns

As predicted, the level of COVID-related concerns indicated in the survey was positively correlated with the percentage of time participants reported spending on TUT related to COVID-19, Spearman’s rho = 0.13, *p* = 0.04.

After demonstrating that greater COVID-related concerns in the survey were associated with greater COVID-related TUT, we then determined whether individuals reporting higher general levels of concern, as indexed by their STAI-6 score, would have more frequent TUTs across all tasks than those with lower levels of concern. This TUT score is the average of the four general TUT scores provided after each task. As predicted, there was a significant positive correlation between these two measures, Spearman’s rho = 0.28, *p* < 0.001, showing that those who reported higher levels of general concern as indexed by the STAI-6 score spent more time thinking about task-unrelated topics during the four attention tasks. These results suggest that heightened latent concerns increase the likelihood of active concerns appearing, as the latent concerns may sometimes spontaneously become active. However, the relatively low correlation value suggests that latent concerns, even when reported at high levels, are frequently inactive. Thus, latent concerns often remain latent, not becoming the subject of conscious thought.

##### Relationship between concerns and performance on attention tasks

**Fig. 4:**
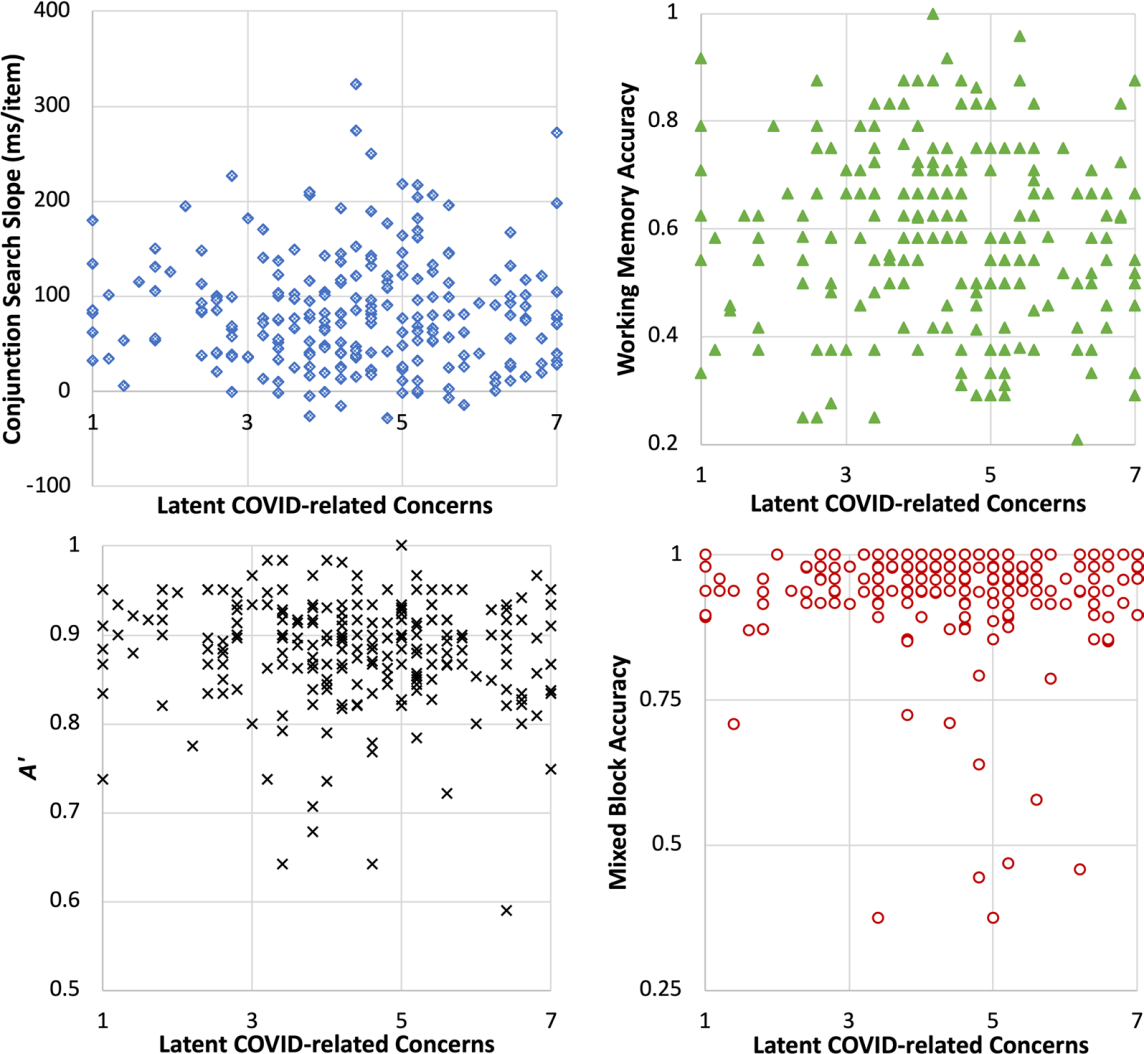
Scatterplot illustrating the lack of correlation between task performance and the pre-task COVID-related concerns (the average of health and financial concerns). Top left: Scatterplot of visual search conjunction search slope versus pre-task COVID-related concerns. Top right: Scatterplot of working memory accuracy versus pre-task COVID-related concerns. Bottom left: Scatterplot of *A′* in scene CPT versus pre-task COVID-related concerns. Bottom right: Scatterplot of mixed-block accuracy in task switching versus pre-task COVID-related concerns

We first tested the correlation between latent COVID-related concerns (measured in the survey) and performance on attention tasks (Fig. 4). Because health concerns and financial concerns are independent contributors to overall COVID-related concerns (Jun et al., 2021), this analysis separately examines the impact of health concerns and financial concerns on attention. None of the measures of performance across the four attention tasks significantly correlated with either health concerns or financial concerns, largest *r* =  − 0.074, *p* = 0.26. All correlations can be found in Table 3. Correlations between survey measures and between all survey measures and attention task performance can be found in Additional file 1: Tables S3 and S4.

**Table 3:** Pearson’s correlation coefficients between survey-indicated concerns and performance on the four attention tasks

	Visual search	Visual working memory	Scene CPT	Task switching
Health concerns	− 0.04	− 0.07	− 0.07	0.02
Financial concern	0.02	− 0.05	− 0.06	− 0.07
Average of health and financial concerns	− 0.01	− 0.07	− 0.08	− 0.03

None of the correlations reached statistical significance

We then tested the correlation between the proportion of TUT reported during each task and performance on that task. All analyses were evaluated with a critical alpha level of 0.0033 to adjust for multiple comparisons. We found a significant negative correlation between accuracy in the mixed blocks of task switching and TUT in that task, Spearman’s rho =  − 0.28, *p* < 0.001. In other words, people who reported more TUT performed worse in task switching. A similar pattern of results was found in the scene CPT. Replicating Jun et al. (2021), scene CPT *A′* negatively correlated with TUT in that task, Spearman’s rho =  − 0.18, *p* = 0.006. Participants who reported more TUT did more poorly in the CPT. The negative correlation between TUT and performance was also observed in the visual working memory task, but this correlation did not reach statistical significance, Spearman’s rho =  − 0.122, *p* = 0.06. A higher TUT did not seem to impair visual search. In this task, participants who reported greater TUT actually demonstrated greater search efficiency in the visual search conjunction or spatial configuration search task (i.e., shallower search slopes), Spearman’s rho =  − 0.19, *p* = 0.003. All correlations can be found in Table 4.

**Table 4:** Spearman’s correlation coefficients between TUT and performance on the four attention tasks

	Visual search	Visual working memory	Scene CPT	Task switching
TUT after task	− 0.19*	− 0.12	− **0.18**	− 0.28*
COVID-specific TUT	− 0.10	− 0.19*	− **0.17**	− 0.21*

Bold indicates uncorrected *p* < 0.01

The negative correlation with visual search slope contradicts predictions of interference from heightened concerns in that task, as greater TUT during visual search was associated with shallower search slope (i.e., more efficient search)

*indicates values significant at the corrected alpha level of *p* < 0.0033

Finally, we performed stepwise regressions for each performance measure that included both active and latent concerns. The regression analysis produced a pattern of results that was consistent with the correlations reported above. For visual search conjunction slope, none of the variables met criteria for inclusion in the regression model. This was also the case for visual working memory capacity. For scene CPT *A′*, the best model included TUT during the scene CPT with a standardized coefficient of − 0.15 (*R*^*2*^ = 0.02, *F*(1, 232) = 5.43, *p* = 0.021). For accuracy in the mixed blocks of task switching, the best model included TUT during task switching with a standardized coefficient of − 0.16 (*R*^*2*^ = 0.03, *F*(1, 232) = 5.90, *p* = 0.016). Thus, greater TUT during tasks that require cognitive control or response inhibition was associated with worse performance, whereas this was not the case in the more perceptual visual search or visual working memory tasks.

### Exploratory analysis on within-task reliability

Finally, we conducted an exploratory analysis on the reliability of each attention task in measuring individual differences. Although many attention tasks produce population-level effects (e.g., steeper search slope in conjunction search than feature search), not all of them produce consistent individual differences. Finding out whether the tasks used in this study produce reliable individual differences is important for interpreting the correlation (or lack of) between these tasks and COVID-related concerns.

To this end, we measured the split-half correlation for the main performance measure for each task. Trials were divided into odd and even trials, and we calculated the average value for each participant for odd and even trials separately. We then ran a two-tailed bivariate Pearson’s correlation between the odd and even trial values.

We observed significant split-half correlations for visual working memory accuracy (*r* = 0.42, *p* < 0.001), scene CPT *A′* (*r* = 0.45, *p* < 0.001), task-switching mixed-block accuracy (*r* = 0.80, *p* < 0.001), and task-switching block management cost (*r* = 0.70, *p* < 0.001). All of these measures indicate strong reliability for individual differences in the indices chosen for visual working memory, scene CPT, and task switching. Therefore, the lack of correlation between COVID-related latent concerns and these measures cannot be attributed to a lack of reliability in the performance measures for these tasks.

Pearson’s correlation for visual search conjunction slope was *r* = 0.05, *p* = 0.46, indicating low individual reliability. As an exploratory analysis, we sought a stronger measure of individual reliability in the visual search task. We identified mean RT in the visual search task as having high individual reliability (*r* = 0.74, *p* < 0.001 in the split-half analysis). Using mean search RT as the reliable measure of visual search performance, we conducted additional exploratory correlational analyses between search RT and the COVID measures. As was the case with conjunction search slope, we observed no significant correlations between visual search RT and COVID-related latent concerns (*r* = 0.09, *p* = 0.20), health-specific latent concerns (*r* = 0.03, *p* = 0.67), or financial latent concerns (*r* = 0.12, *p* = 0.06). Longer search RT was associated with greater TUT during visual search, Spearman’s rho = 0.13, *p* = 0.055, and with greater COVID-specific TUT, Spearman’s rho = 0.115, *p* = 0.08. However, these correlations did not reach statistical significance and were likely spurious given the exploratory nature of the analysis.

## Discussion

Fully understanding the ways in which heightened concerns resulting from the COVID-19 pandemic influence performance on tasks relying on selective or sustained attention requires evaluation of at least three different relationship categories. First, we must understand the relationship between different components of attention. Second, we must understand the distinction between latent concerns, which may be held for long periods of time but may not always be at the forefront of one’s thoughts, and active concerns, which occupy a person’s thoughts and may spontaneously emerge from preexisting latent concerns or may be more acute considerations unrelated to latent concerns. Finally, we may consider the relationship between both kinds of concerns and different components of attention. By testing a large number of participants on a battery of attention tasks, surveying general and COVID-related concern levels, and measuring self-reported TUT, the present study allowed for evaluation of these three relationship categories.

First, the lack of strong correlations between performance measures across the four attention tasks suggests that these tasks tap into different attentional mechanisms, adding to previous findings dividing attention into distinguishable components (Fan et al., 2002; Skogsberg et al., 2015; Treviño et al., 2021). It may be informative to consider a distinction among different attention tasks in terms of whether they primarily rely on perceptual processing or cognitive control. In our preregistration, we hypothesized that working memory and task switching would tap into central attention, or internal attention requiring cognitive control, visual search would tap into perceptual attention, or external attention, and the scene CPT task would tap into both perceptual and cognitive control mechanisms.

The preregistered placement of these four tasks along the continuum from internal cognitive control to external perceptual attention led to three predictions. First, we predicted that if both working memory accuracy and task-switching mixed-block accuracy rely on internal attention, they would positively correlate with each other. Second, we predicted that if the search slope for the conjunction search trials in the visual search task relies almost entirely on external, perceptual attention, it would not correlate with any other task, except for a possible small correlation with scene CPT *A′*. Third, we predicted that if scene CPT *A′* relies to some extent on both internal and external attention, but neither to the extent of the other tasks, it would at most show small correlations with each of the other tasks. The results were largely consistent with these predictions. They indicated that we had selected a broad enough range of tasks to tap into several distinct components of attention that may vary in terms of their relative susceptibility to interference from current concerns.

Second, while the roles of latent and active concerns in driving behavior may be distinct, the two are related. Specifically, higher levels of latent concerns are associated with increased intrusions from active concerns, as seen in the correlation between survey measures of latent concerns and TUT reports of active concerns. We found that individuals reporting higher latent COVID-19 concerns were more likely to have TUTs related to COVID-19 during the attention tasks. This finding replicated the observation of a correlation between survey concerns and TUT in Jun et al. (2021; Spearman’s rho = 0.22). It also aligns with studies showing that negative emotional states due to future-related concerns increase TUT prevalence (Stawarczyk et al., 2013) and that the contents of TUTs often relate to current concerns (McVay & Kane, 2010; Poerio et al., 2013; Smallwood & Schooler, 2015). Similarly, we found that individuals reporting higher general levels of concern, as indexed by their STAI-6 score, had more frequent general TUTs across all tasks than those with lower levels of general concern. Thus, increased latent concerns, both general and COVID-specific, are associated with increased active concerns. It is important to note, however, that the correlations are not large, suggesting that while related, the two concern types are distinct—active concerns may arise in response to external information or spontaneous thoughts independent of existing latent concerns, and latent concerns may not become activated.

Third, active concerns are more likely than latent concerns to interfere with performance on tasks requiring attention. While not all task performance measures correlated with active concern measures—a finding relevant to the following point below—none of the task performance measures yielded measurable correlation with the measures of latent concerns about COVID-19. Future studies that either use stimuli related to the concerns within the cognitive tasks or experimentally “activate” concerns immediately before the cognitive tasks will be important in determining whether latent concerns are more labile when the environment cues them. Nonetheless, the present findings suggest a negligible effect of latent concerns, including those related to COVID-19, on cognitive performance. This does not mean that the pandemic has not influenced mental health and cognitive performance. Rather, concerns about the threats the virus poses do not cause these negative outcomes directly. Importantly, this means that those who remain concerned about and therefore vigilant against the virus are not sacrificing cognitive function unless they are actively engaged in TUT related to those concerns. These data do not support the narrative that continuing to take the virus seriously places individuals in greater danger of being mentally burdened.

Fourth, the extent to which current concerns interfere with attention differs across tasks. We hypothesized that tasks relying on higher-level cognitive control mechanisms would show more interference than tasks relying on perceptual processing. In the preregistration, we assumed that task switching and visual working memory would rely primarily on internal, cognitive control mechanisms, the visual search task would rely primarily on external, perceptual mechanisms, and the scene CPT task would rely somewhat on both cognitive control and perceptual mechanisms. The results partially followed the predictions resulting from these assumptions: As predicted, there was not a negative relationship between TUT and visual search, and performance in task switching decreased as TUT increased, but the same relationship was not significant in the visual working memory task after correcting for multiple comparisons; like task-switching performance, scene CPT *A′* was negatively correlated with TUT.

Thus, task switching showed the susceptibility we predicted, but the visual working memory task did not. It is possible that the visual working memory task does not rely on cognitive control to the same extent as task switching. Although many people consider visual working memory to be an example of attention directed internally (Chun 2011), the task used in the present study may rely more heavily on external, perceptual processing than one may assume. The task requires comparison between internal representations of the encoding display and external stimuli in the testing display, rather than manipulation of information in visual working memory (Pailian & Alvarez, 2020). Brain imaging studies have shown that early sensory areas, such as V1, are involved in retaining visual information in working memory (Harrison & Tong, 2009). Likewise, behavioral studies show that visual working memory is vulnerable to interference from subsequently presented visual stimuli, suggesting that it may have a sensory component like iconic memory (Landman et al., 2003; Makovski & Jiang, 2007; Sligte et al., 2008). These considerations make it likely that both external perceptual processing and internal cognitive control mechanisms contribute to visual working memory.

We may approach the findings from the scene CPT task with similar skepticism about the assumed relative roles of internal and external attentional mechanisms in performance. The negative correlation between *A′* and TUT during the scene CPT task neither confirms nor disproves the hypotheses we outlined in our preregistration. Because we hypothesized that the scene CPT task would rely on both internal cognitive control mechanisms and external perceptual attention, we did not expect a strong correlation but also would not have been surprised to find a correlation. However, recent data suggest that the scene CPT task, with its high required response rate, may primarily rely on cognitive control (Jun & Lee, 2021). Therefore, predictions that assume a high level of reliance on central attention in scene CPT performance may be more valid than those that assume relatively even reliance on perceptual and central attentional mechanisms.

**Fig. 5:**
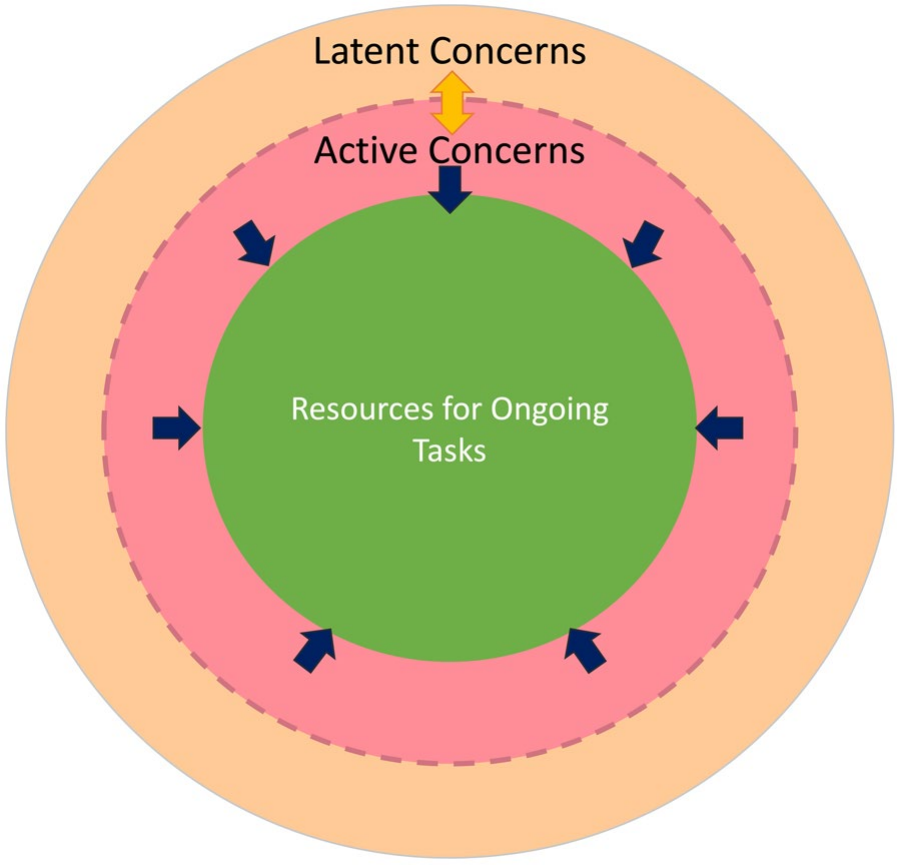
A schematic illustration of the resource contraction theory. Concerns may be latent (orange) or active (pink). Active concerns lead to a reduction in attention to cognitive tasks. Current concerns disproportionately influence tasks that require internal attention (e.g., cognitive control) compared with tasks that rely on external attention (e.g., visual search)

Based on our findings, we propose a resource contraction theory (Fig. 5) that integrates theoretical ideas from diverse perspectives, including dual-task processing, emotion, attention, and the economics of scarcity. First, grounded in basic principles of dual-task processing (Kinchla, 1992; Pashler, 1994), this theory assumes that multiple concurrent tasks compete for attention. The degree of competition is modulated by the similarity between tasks, with greater interference between tasks that share more overlapping cognitive or brain resources (Carpenter et al., 2002).

Second, the resource contraction theory assumes that the internal states of an observer, such as their current concerns, influence the availability of attention for cognitive tasks. This assumption derives from the dual processing framework of emotion (Pessoa, 2009) and the resource depletion account of scarcity (Cannon et al., 2019; Mani et al., 2013; Roux et al., 2015). With the exception of concern-related stimuli, stimuli in ongoing tasks receive reduced processing when levels of current concerns are high. As concerns increase, resources for or time allocated to externally imposed tasks decrease.

Third, the resource contraction theory builds on the taxonomy of attention that divides attention into external and internal attention (Chun et al., 2011). External attention refers to attention directed toward the perceptual world (i.e., perceptual attention, Pashler, 1994). It is involved in tasks such as visual search, texture segmentation, and scene perception. Internal attention refers to attention directed toward an internal representation, such as adherence to complex response rules or resolving response conflict through cognitive control. The resource contraction theory proposes that as a form of internal attention, current concerns disproportionately affect tasks that rely on internal attention, compared to tasks that rely on external attention.

Finally, the resource contraction theory proposes that people have some ability to control the intrusion of current concerns to ongoing tasks. As a result, concerns may take two formats: latent and active. The resource contraction theory assumes that active concerns, not latent concerns, are the main cause of resource contraction. Although many issues induce concerns and people can provide subjective ratings about how worried they are about these issues, most concerns can be kept in a latent format during an externally imposed task. Only active concerns intrude into ongoing tasks. If the concerns are not active at a given moment, they do not intrude into ongoing tasks. Many factors can drive a concern to transition between a latent and an active state. Latent concerns may spontaneously become active. The more severe a latent concern is, the more likely it will spontaneously activate. Conversely, active concerns may become latent when the stake for successful task completion is high. Experimental factors also influence whether concerns are active or latent. For example, an experimenter can activate a concern by asking participants to contemplate a health crisis or financial problem during a task.

In sum, the resource contraction theory provides a useful conceptual framework for understanding how internal states, such as current concerns, influence attention. In this framework, anxiety surrounding the COVID-19 pandemic and the threats it may pose to one’s physical and financial well-being increases the levels of latent concerns in some people. When these concerns are activated, they manifest as TUTs, resulting in a contraction of available resources for ongoing tasks. Such contraction may disproportionately impair internal attention compared with external attention. By assuming that resource contraction results from active rather than latent concerns, this theory also predicts that COVID-related concerns, especially those measured offline, need not always interfere with attention and task performance. The amount of interference will depend on (1) the extent to which COVID-related concerns become active and (2) the degree to which the task relies on internal attention.

The field of cognitive research as it relates to the COVID-19 pandemic has only begun to appreciate the lasting effects of the pandemic on human cognition and behavior. While several studies have explored the lasting cognitive effects of COVID-19 infection, smaller but more widespread effects borne from concerns about COVID-19 could potentially have large effects at the population scale. The present study was crucial in testing this possibility. The finding that overall, concerns about COVID-19 did not interfere with cognition unless thoughts entered conscious awareness as TUT counters the narrative that maintaining a level of caution and concern about the virus is harmful. Being concerned about COVID-19 may reduce the likelihood of contracting the disease without impairing cognitive performance. Yet the present study also underscores the importance of considering both active and latent concerns in such evaluations, as well as a broad range of cognitive tasks that allow detection of effects within some attentional components that may be absent in others.

## Supplementary Information

**Additional file 1.** The full questionnaire, detailed demographic information, and additional correlations and analyses.

## Data Availability

The preregistration for this study will be made available upon publication at https://osf.io/5y9bt/?view_only=615432100a084e2faf535942d1015073. Datasets generated and analyzed in the current study will also be available at that link.

## Footnotes

1 The 15 planned comparisons include three measures of latent concerns (health concerns, financial concerns, and the average of health and financial concerns) and five measures of task performance (conjunction search slope for visual search, visual working memory accuracy, scene CPT *A’*, task-switching mixed-block accuracy, and task-switching RT cost).
