# Supplementary material for: Impact of active and latent concerns about COVID-19 on attention
Source: Cogn Res Princ Implic. 2022 Jun 3;7:48. doi: 10.1186/s41235-022-00401-w (PMC9164188; doi:10.1186/s41235-022-00401-w)
Supplement: Supplementary file 1 — Additional file 1. The full questionnaire, detailed demographic information, and additional correlations and analyses. [file 41235_2022_401_MOESM1_ESM.pdf]

Supplementary Materials

By providing the full survey, these supplementary materials allow for replications of our findings and direct comparisons between this study population and different populations that may be studied in the future. These materials provide comprehensive reports of survey responses and correlations. This presents a more complete picture of the population studied and opens the possibility for new insights to emerge regarding relationships among survey measures.

Table S1. Full Survey.

1. COVID-19 survey

1) Your age (years):

2) Please specify your gender:
    a. Male
    b. Female
    c. Other: ________

3) In which country do you currently reside?

4) In which state do you currently reside? (If you do not reside in the United States, please choose the first option.)

5) Choose one or more races that you consider yourself to be:

   a. White
   b. Black or African American
   c. American Indian or Alaska Native
   d. Asian
   e. Native Hawaiian or Pacific Islander
   f. Other: ________

6) Are you of Hispanic or Latino descent - that is, Mexican, Mexican American, Chicano, Puerto Rican, Cuban, South or Central American or other Spanish culture or origin?

   a. Yes
   b. No

7) What is the highest level of education you completed?

   a. Some grade school
   b. Some high school
   c. High school diploma or GED
   d. Some college or 2-year degree
   e. 4-year college graduate
   f. Some school beyond college
   g. Graduate or professional degree

8) Please indicate your approximated total annual household income before taxes.

   a. Less than $10,000
   b. $10,000 to $19,999
   c. $20,000 to $29,999
   d. $30,000 to $39,999
   e. $40,000 to $49,999
   f. $50,000 to $59,999
   g. $60,000 to $69,999
   h. $70,000 to $79,999
   i. $80,000 to $89,999
   j. $90,000 to $99,999
   k. $100,000 to $149,999
   l. $150,000 or more
   m. I prefer not to answer this question

9) How many members are in your household (including yourself)?

10) Here is a 7-point scale on which the political views that people might hold are arranged from extremely liberal (left) to extremely conservative (right). Where would you place yourself on this scale?

a. Extremely Liberal
b. Liberal
c. Slightly Liberal
d. Neutral
e. Slightly Conservative
f. Conservative
g. Extremely Conservative

11) Each statement below is asking what has changed in your infection history since the coronavirus disease pandemic began. Please check Yes (if you were impacted) or No (if you were not impacted).

	Yes	No
a. Currently have symptoms of this disease but have not been tested.	•	•
b. Previously had symptoms of this disease but never tested.	•	•
c. Tested positive and currently have this disease.	•	•
d. Tested positive for this disease but no longer have it.	•	•

12) Have you received a COVID-19 vaccine?

   a. Yes, I am fully vaccinated
   b. Yes, I am partially vaccinated (I have only received one dose of a two-dose vaccine)
   c. No, I have not received any COVID-19 vaccine

13) Listed below are statements about the potential scenarios that could result from COVID-19 and the pandemic. For each statement, please indicate how concerned you are about that potential scenario (1 = not concerned at all; 7 = extremely concerned).

	1 = I am not concerned at all about this possibility	2	3	4	5	6	7 = I am extremely concerned about this possibility
a. I or people I love may get sick from COVID-19.	•	•	•	•	•	•	•
b. COVID-19 may delay the treatment of other illnesses that I or people I love may have.	•	•	•	•	•	•	•
c. I may lose job-related income due to COVID-19 (i.e., a	•	•	•	•	•	•	•

	pay cut or decrease in business).						
d.	This is a data quality check. Please choose 2.	•	•	•	•	•	•	•
e.	I may lose my job due to COVID-19.	•	•	•	•	•	•	•
f.	Someone that I interact with from outside of my household may infect me with COVID-19.	•	•	•	•	•	•	•

14) The following questions ask about your experiences with social distancing. Social distancing means keeping space between yourself and other people outside of your home.

		None of the days (0 days)	A few days (1-2 days)	Most days (3-4 days)	Every day
a.	In the last 5 days, I have attended social gatherings, outside my home.	•	•	•	•
b.	In the last 5 days, I have gone on shopping trips or outings that were "just for fun."	•	•	•	•

c. In the last 5 days, I have visited nursing homes or long-term care facilities (outside of work duties).	•	•	•	•
d. In the last 5 days, I have been in person-to-person contact with someone who is in a risk group (adults age 50+, people with chronic medical conditions) outside of my household.	•	•	•	•

15) How often are you doing the recommended pandemic hygiene, like washing hands frequently, wearing a face mask, avoiding touching your face, covering coughs, and avoiding frequently touched surfaces in public places?

a. All of the time; I am being extra careful
b. Most of the time; I try my best
c. Sometimes; I do it if I think of it
d. Rarely; I don't worry about these things

16) How would you rate your general health?

a. Excellent
b. Good
c. Fair
d. Poor

17) Has your doctor or any medical provider ever told you that you have any of the following conditions?

a. Heart disease (e.g., congenital heart disease, congestive heart failure, history of heart attack)
b. Hypertension/high blood pressure
c. Diabetes
d. Autoimmune condition
e. Immune deficiency
f. Other underlying health condition ________
g. I do not have any underlying health conditions

18) Since the pandemic started, have you been working in any occupation that requires frequent contact with people from outside of your household? If so, select your occupation below or select "other" and enter the title of your occupation. If not, select "I do not work in an occupation that requires frequent contact with others."

a. Teacher in an in-person classroom
b. Sales clerk at a retail store
c. Grocery store employee
d. Staff working on site at a school with in-person classes
e. Host, waiter, or server at a restaurant
f. Bartender
g. Barista
h. Other ________

   i. I do not work in an occupation that requires frequent contact with others.

19) During the pandemic, how hard has it been for you to pay for the very basic like food, housing, medical care, and heating/cooling? Would you say…
   a. Very hard
   b. Hard
   c. Somewhat hard
   d. Not very hard

20) How would you describe the money situation in your household right now?
   a. Comfortable with extra
   b. Enough but no extra
   c. Have to cut back
   d. Cannot make ends meet

21) How would you describe your job security right now?
   a. Employed with little to no fear of job loss
   b. Employed but concerned about potential job loss
   c. Unemployed but optimistic about ability to find work
   d. Unemployed and unable to find work
   e. Unemployed and not looking for a job

22) Here are a number of statements which people have used to describe themselves. Read each statement and then select the appropriate button to indicate how you feel right now – that is, at this moment. There are no right or wrong answers. Do not spend too much time on any one statement, but give the answer which seems to describe your present feelings best.

	1 = Not at all	2 = Somewhat	3 = Moderately	4 = Very much
a. I feel calm.	•	•	•	•
b. I feel tense.	•	•	•	•
c. I feel upset.	•	•	•	•
d. I feel relaxed.	•	•	•	•
e. I feel content.	•	•	•	•
f. I feel worried.	•	•	•	•

2. Demographic information

Table S2.

Demographic information of participants in the final sample (N=234)

	Frequency	%
Gender		
Male	134	57.26
Female	93	39.74
Other	7	2.99
Race and ethnicity (Participants could choose one or more if applicable)		
White	173	73.93
Hispanic	23	9.83
Asian	40	17.09
Black or African American	23	9.83
American Indian or Alaska Native	1	0.43
Native Hawaiian or Pacific Islander	1	0.43
Other	5	2.14
Highest level of education		
Some grade school	0	0
Some high school	1	0.43
High school diploma or GED	26	11.11
Some college or 2-year degree	88	37.61
4-year college graduate	78	33.33
Some school beyond college	3	1.28
Graduate or professional degree	38	16.24
Political orientation		
Extremely liberal	38	16.24
Liberal	79	33.76
Slightly liberal	47	20.09
Neutral	27	11.54

Slightly conservative	23	9.83
Conservative	12	5.13
Extremely conservative	8	3.42
Annual income before taxes		
Less than $10,000	17	7.26
$10,000 to $19,999	9	3.85
$20,000 to $29,999	14	5.98
$30,000 to $39,999	19	8.12
$40,000 to $49,999	30	12.82
$50,000 to $59,999	21	8.97
$60,000 to $69,999	17	7.26
$70,000 to $79,999	11	4.70
$80,000 to $89,999	17	7.26
$90,000 to $99,999	14	5.98
$100,000 to $149,999	32	13.68
$150,000 or more	25	10.68
I prefer not to answer this question	8	3.42
Household Size (including the participant)		
1	53	22.65
2	45	19.23
3	41	17.52
4	48	20.51
5	32	13.68
6	7	2.99
7	2	0.85
8	0	0
9	1	0.43
Did not answer	5	2.14
Difficulty of paying for the basics like food and medicine		
Not very hard	153	65.38
Somewhat hard	43	18.38

Hard	23	9.83
Very hard	15	6.41
Household financial status		
Comfortable with extra	71	30.34
Enough but no extra	96	41.03
Have to cut back	56	23.93
Cannot make ends meet	11	4.70
Overall health status		
Excellent	55	23.50
Good	149	63.68
Fair	28	11.97
Poor	2	0.85

3. Exploratory analysis on participants' responses to the survey

To understand the relationships among survey items, as well as those between demographics and task performance measures, we conducted pre-registered exploratory correlation analyses between ratings in the demographic questions, task performance across the four attention tasks, ratings in concerns related to COVID-19, general health, COVID-19 infection history, vaccination status, and ratings in compliance with social distancing and hygienic recommendations. The full results of the correlation analyses are presented in Tables S3 and S4.

Pearson's correlation that reached the Bonferroni corrected alpha of $p < .001$ included:

(1) Age: Older participants achieved higher education.

(2) Education: Higher education positively correlated with greater wealth.

(3) Political orientation: More conservative individuals were less concerned about the health-related threats of COVID-19. They were also less likely to engage in social distancing and hygiene behaviors.

(4) Overall health status: Participants who reported themselves as having better general health had greater state anxiety as measured by STAI6 scores.

(5) Infection history: Participants who reported previously experiencing COVID-19 symptoms reported previous positive COVID-19 tests more frequently than participants who did not report previously experiencing symptoms.

(6) Public health guidance: Participants with greater concerns about the health threats of COVID-19 were more likely to report following public health guidance regarding social distancing, mask wearing, hand washing, and other hygienic behaviors.

(7) Measures of latent concerns correlated with one another, with the exception of the correlation between health-specific COVID-related concerns and finance-specific COVID-related concerns, which did not correlate significantly.

(8) None of the attention task performance measures significantly correlated with any of the survey items.

Table S3.

*Pearson's correlation coefficients between responses to the COVID-19 survey and performance on the four attention tasks. Items include <u>demographic</u> questions (1-age, 2-highest level of education, 3-household wealth (income divided by the square root of household size), 4-political orientation, 5-overall health status, 6-history of COVID symptoms, 7-history of positive COVID test), <u>compliance</u> with public health recommendations (8-adherence to social distancing and hygiene guidelines, 9-vaccination status for those tested after vaccines became available), and <u>attention task</u> performance measures (10-search slope in the conjunction trials of the visual search task, 11- percent correct in the visual working memory task, 12-A' in the scene CPT task, and 13-percent correct in the mixed blocks of the cognitive control task). *: p < .001 (Bonferroni corrected alpha). Italics and bold: p < .01 (uncorrected for multiple comparisons).*
Italics: p < .05 (uncorrected for multiple comparisons).

Variables	1	2	3	4	5	6	7	8	9	10	11	12
												Demographic ratings
1. Age	–											
2. Education	**.32 ***	–										

3. Wealth	.03	**.28***	–									
4. Political orientation	*.17*	.00	.07	–								
5. Overall health status (†)	.07	-.11	***-.18***	-.10	–							
6. Previous COVID-19 symptoms	.00	-.03	.09	.06	-.05	–						
7. Previous COVID-19 positive test	-.02	-.01	.04	.02	-.06	**.39***	–					
Behavioral compliance												
8. Distancing and hygiene (†)	.02	.09	***.19***	**.24***	-.05	***.20***	.10	–				
9. Vaccination status	.12	*.25*	.14	-.20	.16	-.17	-.13	.00	–			
Attention task performance												
10. Visual search	.13	.04	-.03	-.05	.03	-.03	-.02	-.09	.05	–		
11. Visual working memory	***-.20***	-.06	.08	-.07	-.03	-.12	.05	***-.19***	-.01	-.06	–	
12. Scene CPT	.00	.09	-.07	-.05	.00	-.04	.05	-.12	-.05	.05	.09	–
13. Task switching (Mixed accuracy)	-.10	*-.15*	-.07	*-.16*	.09	.00	.03	***-.18***	.10	-.09	**.28***	.10

Note: Variables with cross (†) require reverse interpretation, in that higher score in the overall health and behavioral compliance (social distancing and hygienic behavior) represent lower levels of the attribute being evaluated. Not all participants indicated their income or household size, so correlations between wealth and other variables include a sample size of 221. The final 89 participants were the only ones asked about vaccination status, so correlations involving that variable include a sample size of 89.

Table S4.

*Pearson's correlation coefficients among responses to the COVID-19 survey. Items include the same nine demographic questions included in Table S3: demographic questions (1-age, 2-highest level of education, 3-household wealth (income divided by the square root of household size), 4-political orientation, 5-overall health status, 6-history of COVID symptoms, 7-history of positive COVID test), and compliance with public health recommendations (8-adherence to social distancing and hygiene guidelines, 9-vaccination status for those tested after vaccines became available). Because correlations among these nine survey responses were already presented in Table S3, Table S4 will present only correlations between each of those nine measures and survey responses evaluating concerns and anxieties. These include 14-STAI6-score measuring state anxiety, 15-the average of the three health-related items, 16-the average of the two finance-related items, and 17-the average of the five items assessing COVID-related concerns. Because correlations between concerns and attention task performance measures were presented in the main text, they are excluded from Table S4. *: p < .001 (Bonferroni corrected alpha). Italics and bold: p < .01 (uncorrected for multiple comparisons). Italics: p < .05 (uncorrected for multiple comparisons).*

Variables	1	2	3	4	5	6	7	8	9	14	15	16
												Demographic ratings
14. STAI6	***-.19***	-.12	-.10	*-.14*	**.23***	.08	-.07	-.09	.09	–		
15. Health concern	-.07	-.06	-.08	**-.26***	***.20***	.09	-.02	**-.29***	.07	**.35***	–	
16. Financial concern	-.08	-.03	***-.21***	.01	.05	.03	-.10	*-.15*	-.10	**.42***	**.44***	–
17. Average of all five COVID-related concern items	-.08	-.05	*-.17*	*-.16*	*.16*	.08	-.07	**-.26***	.00	**.45***	**.87***	**.83***

Note: Variables with cross (†) require reverse interpretation, in that higher score in the overall health and behavioral compliance (social distancing and hygienic behavior) represent lower levels of the attribute being evaluated. Not all participants indicated their income or household size, so correlations between wealth and other variables include a sample size of 221. The final 89 participants were the only ones asked about vaccination status, so correlations involving that variable include a sample size of 89.
